# Harnessing the power of synthetic data in healthcare: innovation, application, and privacy

**DOI:** 10.1038/s41746-023-00927-3

**Published:** 2023-10-09

**Authors:** Mauro Giuffrè, Dennis L. Shung

**Affiliations:** 1 Department of Medicine (Digestive Diseases), Yale School of Medicine, Yale University, New Haven, CT USA; 2 Department of Medical, Surgical and Health Science, University of Trieste, Trieste, Italy

**Keywords:** Health policy, Medical ethics

## Abstract

Data-driven decision-making in modern healthcare underpins innovation and predictive analytics in public health and clinical research. Synthetic data has shown promise in finance and economics to improve risk assessment, portfolio optimization, and algorithmic trading. However, higher stakes, potential liabilities, and healthcare practitioner distrust make clinical use of synthetic data difficult. This paper explores the potential benefits and limitations of synthetic data in the healthcare analytics context. We begin with real-world healthcare applications of synthetic data that informs government policy, enhance data privacy, and augment datasets for predictive analytics. We then preview future applications of synthetic data in the emergent field of digital twin technology. We explore the issues of data quality and data bias in synthetic data, which can limit applicability across different applications in the clinical context, and privacy concerns stemming from data misuse and risk of re-identification. Finally, we evaluate the role of regulatory agencies in promoting transparency and accountability and propose strategies for risk mitigation such as Differential Privacy (DP) and a dataset chain of custody to maintain data integrity, traceability, and accountability. Synthetic data can improve healthcare, but measures to protect patient well-being and maintain ethical standards are key to promote responsible use.

## Introduction

Data is the lifeblood of modern healthcare bearing the potential to improve patient care by powering clinical research, and advancing public health initiatives. However, the promise of real-world data to enable personalized medical care, to guide policymaking, and to respond to rapidly changing conditions is tempered by the significant challenges inherent in accessing high-quality datasets. Synthetic data offers an attractive alternative that addresses privacy concerns, streamlines data utility agreements, protocol submissions, and ethics review approvals, and decreases costs. In light of this potential, synthetic data has been utilized in other fields such as finance and economics to evaluate risk management, to optimize complex portfolios, and to enhance algorithmic trading particularly when access to real-world financial data is limited or privacy concerns are raised by financial agencies^1^. However, the use of synthetic clinical data to evaluate risk in clinical decision-making is modulated by the following challenges: the liability with modeling error is more consequential and the stakes for patients and providers are high^2,3^. Healthcare stakeholders are typically risk-averse and prefer precise replication of the original healthcare records and have decreased trust in clinical diagnosis derived from AI-based prediction models using synthetic datasets^3,4^. In this article we review the generation techniques, applications, and definitions of synthetic data in the context of healthcare and explore associated privacy issues. We highlight these concerns and propose strategies to mitigate harm, aiming to harness the full potential of synthetic data in advancing medical research and patient care.

## What is synthetic data?

Despite the interest in synthetic data, the field is still developing and has no clear consensus regarding a unified definition. The absence of a universally accepted definition of synthetic data leads to inconsistent use of the term and interpretations that vary across contexts, thereby affecting reproducibility and transparency in research involving synthetic data. A working definition has been recently proposed by the Royal Society and The Alan Turing Institute, where synthetic data is “data that has been generated using a purpose-built mathematical model or algorithm, with the aim of solving a (set of) data science task(s)”^5^. This proposed definition is particularly interesting when compared to the alternative definitions emphasizing the creation of new data values mirroring the original data’s statistical characteristics^6^. This new working definition stresses the functional and intentional aspects of synthetic data, shifting focus from mere replication of statistical properties to the strategic use of these artificial constructs in addressing complex scientific challenges. The main types of synthetic data in clinical settings include tabular, time-series, or text-based synthetic data. Additional categories also include synthetic images, video, or audio simulation.

### Synthetic data generation and types

The concept of using synthetic data, originating from computer-based generation, to solve specific tasks is not novel. This approach traces back to the foundational work of *Stanislaw Ulam* and *John von Neumann* in the 1940s, focusing on Monte Carlo simulation methods^7^. Modern synthetic data generators range from deep learning structures like Generative Adversarial Networks (GANs)^8^ and Variational Auto-encoders (VAEs)^9^, to agent-based econometric models^10^, or stochastic differential equations simulating physical or economic systems^11^. The growing interest in the medical use of synthetic data has led companies to develop open source (e.g., Synthea)^12^ or commercial (e.g., MDClone’s Synthetic Data Engine)^13^ tools that can automate the generation of high-quality and clinically realistic synthetic datasets.

Regardless of the methodology, the classification of synthetic data is not rigid and often represents a spectrum that ranges from partially to fully synthetic. Partially synthetic data incorporates real-world data with synthetic data^14^. In healthcare, partially synthetic data has been used as a proxy for real-world data to protect patient privacy while still allowing researchers to conduct analyses. For example, Loong et al.^15^ investigated the use of partially synthetic data as a proxy for real-world data in large-scale health surveys. The study reviewed inferential methods for partially synthetic data, including the selection of high disclosure risk variables for synthesis, specification of imputation models, and identification of disclosure risk assessment. The results showed that partially synthetic data can be used to protect patient privacy while still allowing researchers to conduct analyses.

Fully synthetic data is created entirely de novo based on predefined rules, models, or simulations^16^. It does not rely on or represent real-world data but is instead designed to replicate the kinds of complexity and variability that might be seen in real-world scenarios. Other than data quality, fully synthetic data can address the issue of data scarcity that may affect both linear and non-linear models as well as the rapidly proliferating large language models by creating supplementations to the available datasets.

## Applications of synthetic data in healthcare

Synthetic data has the potential to estimate the benefit of screening and healthcare policies, treatments, or clinical interventions, augment machine learning algorithms (e.g., image classification pipelines), pre-train machine learning models that can then be fine-tuned for specific patient populations, and improve public health models to predict outbreaks of infectious diseases^17–27^.

Building upon the potential uses of synthetic data mentioned earlier, the study by Davis et al.^28^ serves as a tangible example of how these datasets can be generated and utilized to investigate healthcare policy implications, particularly in the context of demographic aging. The authors explored the use of micro-simulation techniques to create a synthesized dataset and test policy options, focusing on the case of health service effects under demographic aging. The study integrates multiple data sources, including the New Zealand Health Survey (NZHS), Australian Health Survey (ANHS), and National Primary Medical Care Survey (NPMCS) to create a synthetic dataset for microsimulation in healthcare policy analysis. The process involved imputation techniques to enrich the representative sample from NZHS with data from ANHS and NPMCS. Subsequently, the synthesized dataset could be used to evaluate the impact of various policy scenarios on health service outcomes, such as variations in morbidity and disability, community support, and doctor behavior. These scenarios are presented as binary alternatives, enabling the exploration of both optimistic and pessimistic policy outcomes on healthcare demand and resource utilization. Furthermore, the authors analyzed the impact of demographic aging by re-weighting the 2002 population to a 2021 projection based on medium birth, mortality, and migration rates. The average number of visits increased slightly from 6.7 to 6.9 per year for general practitioner (GP) users. The researchers tested three components: morbidity, social support, and practitioner behavior. For the 65+ age group, average GP visits doubled from 8.8 to 15.3 for higher morbidity scenarios, while social support remained unaffected. The authors also found that prescription rates for more interventionist GPs were nearly double compared to least interventionist colleagues. Referral rates for the most interventionist GPs were six times higher than the most conservative (30% vs. 5%). The use of synthetic data in this example is an approach that could allow policymakers to make decisions on resource allocation after analyzing a range of possible scenarios to gain insights into healthcare demand and resource utilization.

The broad applicability and adaptability of synthetic datasets extend beyond their use in policy simulation and across multiple data modalities. A different but equally innovative approach to using synthetic data is illustrated in a study by Julia et al. in 2020 leveraged a Natural Language Processing (NLP) model by training it with synthetic datasets created from patient discharge reports^29^. The model effectively targets mental health diseases to predict the corresponding diagnosis and phenotypes. Electronic Health Records (EHRs) provide NLP data that can be utilized for specifying the critical aspects of a patient’s disease and anticipated pathways. For patients with mental health issues, the EHRs are typically based on an unstructured text that can be generated synthetically for training language models to classify complex diseases. Since mental health information is considered particularly sensitive, the use of synthetic text mitigates the risk for compromising sensitive information for individual patients. Synthetic datasets can also be useful in clinical challenges that involve large populations and epidemiological phenomenon, such as in the COVID-19 pandemic. Synthetic datasets were useful in improving the challenge of improving data scarcity in augmenting data volume in imaging studies in the COVID-19 pandemic^30^. In particular, Das et al.^31^ created conditional synthetic datasets for chest CT scans to classify COVID-19 patients from a population of normal individuals and pneumonia patients. The authors demonstrated that using synthetic datasets could improve the accuracy COVID-19 detection process compared to the original datasets. The discussed studies illustrate the multifaceted potential and adaptability of synthetic data in healthcare. Whether for probing healthcare policy impacts in an aging demographic, enhancing NLP models for mental health diagnoses, or bolstering COVID-19 detection in CT scans, synthetic datasets provide invaluable tools for researchers. By bolstering the volume and variability of available data, synthetic datasets facilitate more robust and comprehensive analyses, thereby informing and improving healthcare strategies, machine learning models, and disease detection methods.

### Synthetic data and digital twins

Synthetic data is also helpful for digital twins, which are virtual replicas of physical systems or processes that can be used to simulate and predict their behavior in real time. The use of digital twins in healthcare is still underdeveloped, but synthetic data could be used to create personalized models of patients, needed to optimize treatment plans, and improve patient outcomes. One area where digital twins are being increasingly used is in hospital efficiency and operations, where synthetic data is used to create realistic models that can simulate different scenarios and predict outcomes. In the creation of a hospital digital twin, synthetic data can be used to simulate different scenario, such as changes in patient volume, staff level of training, and equipment availability, thus hypothetically allowing administrators to optimize staffing levels and resource allocation, reducing cost and improve patient outcomes^32,33^. Synthetic data can also be used to create digital twins of patients and their trajectories, which can be used to optimize treatment plans and improve patient outcomes^34–37^. By creating a personalized model of a patient, synthetic data can be employed to simulate different treatment options and predict their effectiveness, thus improving patient outcomes and possibly reducing costs.

### Potential pitfalls: bias and interpretability

The incorporation of synthetic data in healthcare has been lauded for its potential to circumvent the challenges surrounding data scarcity and privacy. However, this potential carries concerns such as the risk of bias amplification, low interpretability, and an absence of robust methods for auditing data quality.

Bias is defined as a systematic discrepancy or persistent deviation that originates during the data sampling or testing process. This can lead to an overestimation or underestimation of the risks associated with specific clinical outcomes. Consequently, if the primary dataset used to generate synthetic data carries inherent biases, the synthetic data could unintentionally magnify these biases. This can result in misinformed or discriminatory outcomes in medical research and practice, further perpetuating existing inequalities and placing vulnerable populations at heightened risk of harm and discrimination^38,39^. This bias could originate from the population under study, the techniques employed to collect data, or the methods used to derive the new dataset^40–42^.

For instance, if a synthetic dataset is trained on a dataset of facial images that majorly includes people from a certain ethnicity, the synthetic images generated will naturally reflect this imbalance, thus perpetuating the initial bias. This leads to AI systems being “blind” to data beyond their training sets, unable to accurately represent or make fair decisions about the unrepresented categories. This is known as the “out-of-distribution” (OOD) problem, a key challenge for AI systems working with synthetic data^43^. Despite synthetic data may potentially solve this limitation by oversampling under-represented characteristics, the danger lies in the risk of overgeneralization and potential creation of non-existent or incorrect correlations. This artificial augmentation may in fact worsen the out-of-distribution issue, by falsely representing certain demographics and their associated medical profiles. Hence, it is crucial that the generation of synthetic data is accompanied by scrutiny and consistent evaluations to minimize these biases. Automatic AI-based methods can be used to address the OOD problem, such as the incorporation of anomaly detection techniques which can identify instances that deviate significantly from the training data distribution, helping to detect and handle OOD examples^44^. By flagging or rejecting such examples, AI systems can avoid making unreliable predictions or decisions.

Bias can also be derived from the generative methods used to derive the synthetic dataset. There are considerable challenges associated with interpreting synthetic data generation models, including the black-box nature of generation algorithms, limitations in the evaluation metrics, and the potential for overfitting or underfitting. This lack of transparency can erode trust in the generated synthetic data, making it difficult for healthcare professionals and researchers to make reliable conclusions or informed decisions based on the data^45^. Explainable AI (XAI) techniques play a crucial role in ensuring the interpretability and transparency of AI systems, particularly when dealing with synthetic data. XAI methods enable users to understand the underlying mechanisms and decision-making processes of AI models, providing insights into the input-output relationships and the presence of biases^46^. In the context of healthcare, XAI techniques such as SHAP (SHapley Additive exPlanations) have been used to interpret the predictions made by machine learning models, ensuring transparency and accountability in decision-making^47^. XAI methods allow users to scrutinize and understand the decisions made by AI systems, which is particularly crucial in domains where decision-making should be transparent, such as healthcare^47^. In the context of synthetic data, XAI techniques can help assess if the synthetic data maintains the desired input-output relationships similar to those found in real data^46^. By using XAI methods, it becomes possible to identify biases and assess the extent to which the synthetic data represents real-world scenarios. However, it is important to note that XAI methods are not without challenges. The interpretability of AI models and the explanations provided by XAI techniques can be subjective and context-dependent^48^. Different stakeholders may have different requirements and interpretations of what constitutes a satisfactory explanation^48^. Additionally, the trade-off between accuracy and interpretability should be carefully considered, as more interpretable models may sacrifice some level of predictive performance^49^.

A significant challenge that arises in the realm of synthetic data pertains to the necessity for robust auditing methods, particularly when XAI methods prove insufficient in evaluating the accuracy and representativeness of the data. The crux of the issue lies in the fact that conventional techniques used to generate synthetic data may inadequately capture the rich complexity and diverse array of real-world medical scenarios. Consequently, the development of novel auditing methods becomes imperative to ensure the true representativeness of synthetic data. One potential approach to auditing methods involves leveraging advanced statistical techniques and machine learning models to accurately assess the similarity between synthetic and real-world datasets. Techniques such as distribution matching^50^, correlation analysis^51^, and dimensionality reduction^52^ can capture the intricate correlations and patterns inherent in real-world medical scenarios, enhancing the data’s representativeness.

Another strategy would involve creating domain-specific evaluation metrics and benchmark datasets, particularly tailored for healthcare applications. By curating benchmarks that accurately represent a wide spectrum of real-world medical scenarios, researchers and practitioners can effectively compare the performance of various synthetic data generation techniques^53^. It’s also important to involve patients and healthcare professionals in the development and validation of synthetic data to ensure its relevance and representation of real-world medical scenarios. By involving human input, AI systems can learn from the expertize and domain knowledge of humans to improve their performance. This human-in-the-loop approach can help address the challenges posed by synthetic data scenarios and enhance the reliability and robustness of generative models^54^.

Lastly, transparency in the form of clear documentation of the data generation process, potential limitations, and data biases can help identify actual and potential errors. In balancing the benefits of synthetic data with the challenges of bias, interpretability, and the need to audit data quality, it becomes crucial to prioritize patient well-being and maintain ethical standards in healthcare.

## Privacy concerns and regulatory agencies

Synthetic data poses serious risks to data privacy and protection. As argued in a recent scoping review^55^, privacy is not something to be considered on as an afterthought once a system has already been designed and deployed; a “privacy-by-design” mindset should proactively be applied, particularly when working with clinical data. The key challenge is to ensure that synthetic data derived from sensitive medical information does not unintentionally disclose identifiable details about individuals or lead to re-identification, violating privacy and data protection principles.

### Regulatory blind spots and proposals

At present, there is no clear legislation surrounding the use of synthetic data^56^ and current data protection regulations such as the General Data Protection Regulation (GDPR) and the Health Insurance Portability and Accountability Act (HIPAA) are limited in their ability to address all the potential risks associated with synthetic data^57^. The two sets of regulations follow the principle that all patients must give their consent before their data are processed or shared, with exceptions when data processing is mandatory, such as for payment or treatment purposes. The simplest method of privacy protection is to remove all fields that could directly and uniquely identify an individual as recommended by HIPAA, which identified 18 such items (e.g., name, social security number, phone number). This was deemed sufficient to de-identify data until the middle of the 2000s, and following the HIPAA Privacy Rule 45 CFR 164, such data is no longer considered Protected Health Information (PHI)^58^. The underlying premise was that the disclosure did not pose a material risk to privacy once these 18 types of information were deleted, and the disclosing entity did not actually know that the data in the de-identified dataset could be used to identify an individual. However, it has recently become evident that a variety of other data fields can be used to identify people^59^. GDPR expands the scope of protected information beyond the definition of PHI, using the term “personal data”, which relates to any information related to an identified or identifiable natural individual. While HIPAA is limited to information generated by healthcare providers related to the medical treatment of patients, the GDPR refers to a natural individual as an entity that can be identified directly or indirectly by other indicators such as physiological, genetic, mental, and cultural identity^58^. The GDPR delineates two forms of data de-identification: pseudonymization and anonymization. Data pseudonymized under GDPR, which is still subject to its legal constraints, often resembles de-identified data under HIPAA. Encrypted with a patient key, data could be de-identified under HIPAA, but it would only be pseudonymized under GDPR, and thus still subject to its regulations. Full anonymization under GDPR is hard to achieve and offers minimal analytical value. Using GDPR-protected data necessitates substantial legal compliance, especially for non-EU entities^60^. Identifying what constitutes “identifiable” data is complicated by re-identification potential. Recent studies have disproved the that de-identification definitively prevents re-identification, citing examples of predictability using accessible personal details. While the challenges of synthetic data regulation are not confined to the field of medical research alone, they also permeate into other facets of healthcare, like the development of medical devices. The work by Chen et al.^61^ sheds light on this broader perspective, underscoring the crucial role of regulatory bodies in facilitating the judicious application of synthetic data in healthcare, specifically within the arena of medical devices incorporating artificial intelligence and machine learning algorithms. The Artificial Intelligence Synthetic Data for Medical Devices (AISAMD) program, a joint venture between the US Food and Drug Administration (FDA) and Standards and Technology (NIST), endeavors to create a framework for the utilization of synthetic data for evaluating such medical devices^62^. This initiative underscores the potential of synthetic data in machine learning for medical applications while stressing the need for stringent validation methods and guidelines for responsible usage in regulatory submissions. This regulation of synthetic data, bears implications not just for patient privacy and data security, but also for the advancement of technological innovation in healthcare. However, incorporating the ethical and legal challenges^63^ associated with synthetic data sharing and usage often straddle the definitions set by existing data protection regulations, thereby creating a regulatory blind spot. This loophole could potentially be exploited by malicious entities, leading to discriminatory use of synthetic data and exacerbating health disparities, undermining efforts to improve patient outcomes. Given this regulatory ambiguity, it behooves systems to revise current data protection legislation with precise definitions and more stringent controls for synthetic data. The proposed measures include updating data protection regulations to encompass synthetic data, establishing a centralized regulatory body for oversight, and promoting transparency and accountability in the development and application of synthetic data. Additionally, it is equally important that future research continues to explore potential privacy risks associated with synthetic data and devise strategies for mitigating these risks. This research should also scrutinize the technical, social, and ethical implications of synthetic data usage in healthcare, offering a holistic examination of this emergent field.

#### The opportunity of differential privacy

Brauneck et al.^63^ recently reviewed privacy-enhancing technologies (PETs) from a legal standpoint to engage in a thoughtful discussion of how GDPR legislation in the European Union (EU) relates to commonly used PETs including federated learning (FL), secure multiparty computation (SMPC), and differential privacy (DP). DP a concept first proposed in 2006 by Dwork et al.^64^, is gaining broad acceptance as a solid, practical, and trustworthy privacy framework and its application has been also explored with synthetic data^65–67^. DP is a precise mathematical constraint that ensures the privacy of individual pieces of information in a database while answering queries about the aggregate. The concept of DP is based on the notion of adding noise to the data to protect the privacy of individuals. This noise introduction is governed by two parameters—epsilon and delta. Epsilon is a parameter that controls the amount of noise added to the data by varying the privacy budget, which represents the maximum amount of privacy loss that can be tolerated. A smaller value of epsilon indicates a higher level of privacy protection, but it also results in a higher level of noise in the data. Delta is a parameter that controls the probability of a privacy breach. It is a measure of the probability that the privacy of an individual is compromised. A smaller value of delta indicates a lower likelihood of a privacy breach but also results in higher noise in the data. The choice of the Epsilon parameter to achieve a balance between privacy and statistical utility may result difficult, with researchers proposing the institution of an “Epsilon Registry” to help make informed implementation choices^68^. As argued by Ficek et al.^69^ despite its robust protections, DP has not yet seen widespread adoption in the health sector. The focus so far has been primarily on creating predictive algorithms, sanitized data publishing, and training machine learning algorithms, particularly in the areas of genomics, neuroimaging, and personal device-derived health data streams. However, significant gaps are crucial for epidemiology and clinical research, especially for explanatory modeling and statistical inference. Another challenge is the privacy-utility trade-off; hence, experimental deployment and real-world case studies are required to understand this better.

Bridging this gap between the theoretical robustness of differential privacy and its practical implementation in the health sector, groundbreaking efforts such as the one by Jordon et al.^70^ have started to emerge, utilizing differential privacy in the context of synthetic data generation. Their novel approach incorporates a modification to the Private Aggregation of Teacher Ensembles (PATE) methodology, incorporating it into GANs for the creation of privacy-preserving synthetic data. Named PATE-GAN, this system utilizes a generator network for synthetic data production from random noise, which is then appraised by a discriminator network. This innovative approach replaces the single discriminator network with multiple teacher networks. Each network is trained on a separate segment of the initial dataset and provides feedback to the generator. This aggregation ensures a balance in model influence and upholds robust differential privacy. PATE-GAN has proven superior to conventional, yet outdated benchmarks, such as Differential Private-GAN (DP-GAN)^71^ and excels in generating synthetic data that closely resemble the original dataset. To evaluate the similarity of synthetic samples with the original data, the authors propose a new metric, termed “synthetic similarity.” This cutting-edge technique offers enhanced performance and a unique evaluation metric, making it adaptable across a variety of datasets and applications. In essence, PATE-GAN constitutes a significant stride forward in the realms of machine learning and privacy. In summary, PATE-GAN, through its innovative approach to differential privacy and the introduction of a unique “synthetic similarity” metric, represents a major advancement in the intersection of machine learning and data privacy, enabling the production of high-quality synthetic data that balances privacy protection with utility across various datasets and applications.

In conclusion, as privacy-enhancing technologies evolve to meet the challenges posed by data privacy regulations and the need for practical implementation in health research, differential privacy emerges as a robust, reliable, and promising approach. Despite its current limitations, particularly in the context of explanatory modeling and statistical inference, its adoption in cutting-edge frameworks such as PATE-GAN highlights its versatility and potential. PATE-GAN’s innovative approach, the introduction of the “synthetic similarity” metric, and the ability to generate high-fidelity synthetic data show a significant advancement in the delicate balance between data privacy and utility.

### Establishing a digital chain-of-custody

Despite the promise of DP to mitigate risk, it is not a panacea. The healthcare community must consider safeguards on the diffusion of synthetic datasets by developing and implementing appropriate regulations. For example, it is crucial to establish a robust *digital* *chain of custody* to ensure the integrity, security, and privacy of data throughout its lifecycle and it must encompass data sharing, storing, and disposal to provide transparency, traceability, and accountability at each stage.

The application of a digital chain of custody in data sharing is crucial for ensuring the integrity, security, and accountability of shared data. The digital chain of custody refers to the process and documentation that tracks the custody and movement of data from its collection to its final destination^72,73^. Digital chain of custody applications and software have been developed to document and track digital evidence, ensuring its integrity and providing a verifiable record of its custody^72,73^. Blockchain technology has also been utilized to enhance the chain of custody in data sharing. Blockchain-based systems provide a decentralized and immutable ledger that records the transactions and movements of data^74^. For example, Wang et al.^75^ proposed a chain-of-custody system is needed to document the sequence of custody of sensitive big data. They designed a prototype of a block-chain big-data sharing system (BBS), where a user can register a dataset with BBS for sharing. To acquire the shared file an authenticated and authorized recipient must use transactions and interacts with BBS across four stages including file transfer request, encrypted data transfer, file key retrieval, and file decryption. Each transaction is recorded in the ledger and serve as chain of custody to document the trail of the data.

This example, among others, has been previously employed to share real-world data, but there is no current process in place for synthetic datasets. The digital chain of custody should begin with generating synthetic data, outlining the methodologies and techniques used to create the data, as well as any privacy-preserving measures implemented, such as differential privacy. This information should be documented and accompany the synthetic data as it moves through various stages of its lifecycle. During data sharing, a secure and controlled process should be established for transferring synthetic data between different parties, such as healthcare providers, researchers, and policymakers. This process should include the use of encryption, secure authentication, and access control mechanisms to protect the data from unauthorized access or tampering. Furthermore, each data-sharing transaction should be logged, detailing the data transfer’s sender, receiver, timestamp, and purpose. For data storage, the chain of custody should ensure that synthetic data is securely stored using encryption and access control mechanisms to protect it from unauthorized access, modification, or leakage. Data storage locations and associated security measures should be documented and regularly audited to confirm compliance with established data protection standards and regulations. Finally, when it comes to data disposal, a clear protocol should be in place for the secure deletion or destruction of synthetic data once it is no longer needed. It involves using secure erasure methods to prevent the recovery of deleted data and maintaining disposal records that detail the time, method, and reason for data destruction.

In addition to these measures, it is vital to implement regulations that mandate a comprehensive research proposal evaluation before granting access to synthetic data. It ensures that the research plan is robust, genuine, and has a clear data analysis objective. Furthermore, researchers should be required to provide timed-release updates on the progress of their analysis and usage of the synthetic data, fostering transparency and allowing for better monitoring and evaluation of the data’s impact. Implementing these regulatory requirements makes it possible to mitigate potential risks associated with the misuse of synthetic data while promoting responsible research practices and maximizing the benefits of synthetic data in healthcare.

## Discussion & conclusion

**Fig. 1:**
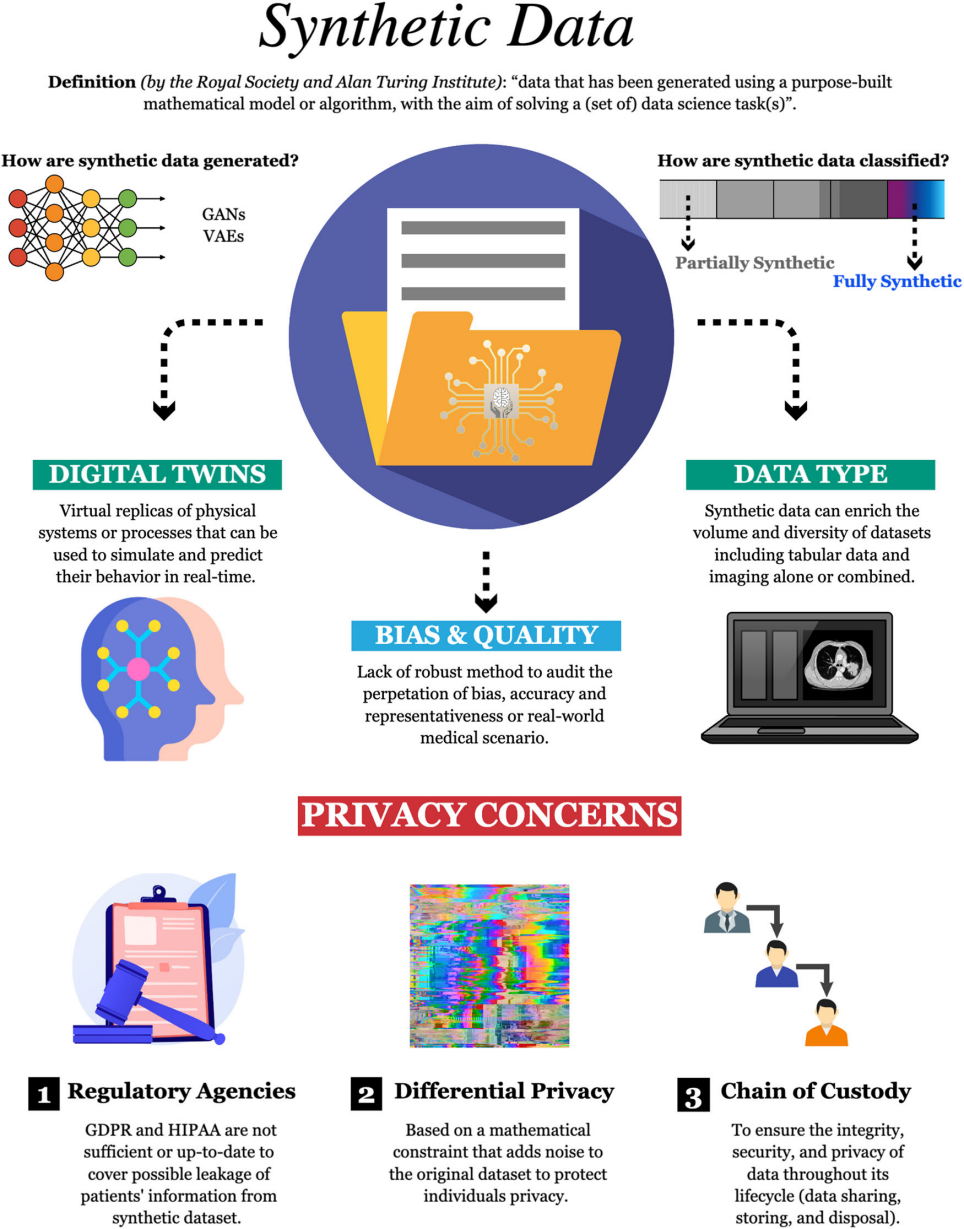
Highlights on Synthetic Data and their application in healthcare research, reviewing bias, quality, and privacy concerns. Despite the first attempt to generate synthetic data dates bate to the 1940s, current state of the art methods use Generative Adversarial Networks (GANs) or Variational Auto-encoders (VAEs). In terms of classification, synthetic data encompasses a spectrum that ranges from partially to fully synthetic. While partially synthetic incorporates real-world data, fully synthetic data are generated de novo. As reported in the text, synthetic data can have several applications in healthcare research, including imaging, infective disease prevention and outbreaks prediction, and digital twins. However, the lack of robust methods to audit the perpetration of bias, accuracy, and representativeness of real-world medical scenarios, has severely limited interpretability, use and trust from the healthcare sector. One of the greatest concerns related to synthetic data involves patients’ privacy. Current regulations from General Data Protection Regulation (GDPR) and the Health Insurance Portability and Accountability Act (HIPAA) are not sufficient or up-to-date to cover possible leakage of patients’ information from synthetic dataset. In this context, differential privacy may result valuable, but its usage has been limited by the privacy-utility trade-off. The definition of a clear chain of custody, can ensure integrity, security, and data privacy throughout data lifecycle providing transparency, traceability, and accountability at each stage.

AI applications in medicine can improve research capabilities and create cost-effective solutions but require careful consideration of potential biases in synthetic data to ensure accurate representation and adequate safeguards for privacy such as differential privacy and a digital chain of custody. As summarized by Fig. 1, the creation and application of synthetic data should be tailored to specific use cases. In healthcare, significant privacy concerns underscore the need for the implementation of privacy-preserving techniques to protect sensitive medical data. We propose the concept of a digital chain of custody for synthetic data to ensure data integrity, security, and confidentiality throughout its lifecycle. To navigate these intricacies, collaborative efforts between the healthcare community and regulatory agencies are imperative in developing and implementing laws and regulations governing synthetic data. Moreover, to establish comprehensive guidelines and best practices for the responsible use of synthetic data in medicine and healthcare, it is crucial for healthcare providers, researchers, technology developers, and patients to be actively engaged and collaboratively involved.

In conclusion, while synthetic data possess the potential to revolutionize healthcare by offering improved research capabilities and cost-effective solutions, overcoming the challenges related to biased information, data quality concerns, and potential privacy risks are of paramount importance. This calls for the healthcare community’s active participation in discussions and collaborations with regulatory bodies, technology developers, and patients, thus fostering a proactive approach to harness the transformative power of synthetic data. Such a process will prioritize patient well-being and uphold ethical standards in medicine and healthcare.

## Data Availability

Not Applicable.
